# Identifying biomarkers deciphering sepsis from trauma-induced sterile inflammation and trauma-induced sepsis

**DOI:** 10.3389/fimmu.2023.1310271

**Published:** 2024-01-12

**Authors:** Praveen Papareddy, Michael Selle, Nicolas Partouche, Vincent Legros, Benjamin Rieu, Jon Olinder, Cecilia Ryden, Eva Bartakova, Michal Holub, Klaus Jung, Julien Pottecher, Heiko Herwald

**Affiliations:** ^1^ Division of Infection Medicine, Department of Clinical Sciences, Lund University, Lund, Sweden; ^2^ Genomics and Bioinformatics of Infectious Diseases, Institute for Animal Genomics, University of Veterinary Medicine Hannover, Hannover, Germany; ^3^ Hôpitaux Universitaires de Strasbourg, Service d’Anesthésie-Réanimation & Médecine Péri-opératoire - Université de Strasbourg, Fédération de Médecine Translationnelle de Strasbourg (FMTS), Strasbourg, France; ^4^ Département d’Anesthésie-Réanimation et Médecine Peri-Operatoire, Centre Hospitalier et Universitaire (CHU) de Reims, Université de Reims Champagne-Ardenne, Reims, France; ^5^ Réanimation Médico-Chirurgicale, Trauma Center, Pôle Médecine Péri-Opératoire, Centre Hospitalier et Universitaire (CHU) de Clermont-Ferrand, Clermont Ferrand, France; ^6^ Division of Infection Medicine, Helsingborg Hospital and Department of Clinical Sciences Helsingborg, Lund University, Helsingborg, Sweden; ^7^ Department of Infectious Diseases, First Faculty of Medicine, Charles University and Military University Hospital Prague, Prague, Czechia

**Keywords:** sepsis, trauma, bacteremia, systemic inflammatory response syndrome, biomarkers, A2M, IL1F10, SYT13

## Abstract

**Objective:**

The purpose of this study was to identify a panel of biomarkers for distinguishing early stage sepsis patients from non-infected trauma patients.

**Background:**

Accurate differentiation between trauma-induced sterile inflammation and real infective sepsis poses a complex life-threatening medical challenge because of their common symptoms albeit diverging clinical implications, namely different therapies. The timely and accurate identification of sepsis in trauma patients is therefore vital to ensure prompt and tailored medical interventions (provision of adequate antimicrobial agents and if possible eradication of infective foci) that can ultimately lead to improved therapeutic management and patient outcome. The adequate withholding of antimicrobials in trauma patients without sepsis is also important in aspects of both patient and environmental perspective.

**Methods:**

In this proof-of-concept study, we employed advanced technologies, including Matrix-Assisted Laser Desorption/Ionization (MALDI) and multiplex antibody arrays (MAA) to identify a panel of biomarkers distinguishing actual sepsis from trauma-induced sterile inflammation.

**Results:**

By comparing patient groups (controls, infected and non-infected trauma and septic shock patients under mechanical ventilation) at different time points, we uncovered distinct protein patterns associated with early trauma-induced sterile inflammation on the one hand and sepsis on the other hand. SYT13 and IL1F10 emerged as potential early sepsis biomarkers, while reduced levels of A2M were indicative of both trauma-induced inflammation and sepsis conditions. Additionally, higher levels of TREM1 were associated at a later stage in trauma patients. Furthermore, enrichment analyses revealed differences in the inflammatory response between trauma-induced inflammation and sepsis, with proteins related to complement and coagulation cascades being elevated whereas proteins relevant to focal adhesion were diminished in sepsis.

**Conclusions:**

Our findings, therefore, suggest that a combination of biomarkers is needed for the development of novel diagnostic approaches deciphering trauma-induced sterile inflammation from actual infective sepsis.

## Introduction

Trauma and sepsis are significant healthcare problems that urgently require novel diagnostic methods to enable optimal and timely medical care (1, 2). This is all the more true in trauma patients. In both acute conditions, septic shock, and trauma patients may be admitted to the intensive care unit (ICU) for initial treatment and stabilization and cared for by the same multisciplinary intensive care teams. At this stage clinicians are urged to choose the correct treatment including eradication of microbial foci and provision of antimicrobials for the septic but to refrain from such treatment in trauma patients (3). Indeed, inappropriate surgical intervention for foci removal in trauma patients may exacerbate inflammation (4) while indiscriminate use of antibiotics may promote the emergence and colonization of multidrug resistant bacteria as well as leading to wellknown side effects of such treatment, especially with broadspectrum antibiotics. Clinical score systems and routine blood sample assays may direct the clinician but there is yet no specific marker to make this distinction and thus, appropriate care remains challenging as even gene expression is very close in both conditions (5). First, trauma patients may develop profound immunosuppression making them more susceptible to severe infection (6). Second, due to the early release of Damage-Associated Molecular Patterns (DAMPs) (7), severe trauma patients develop systemic inflammation, hyperthermia, vasoplegia (8), hyperleucocytosis, and elevated inflammatory biomarkers all mirroring sepsis despite no infection (9, 10). The same release of DAMPs also promotes immune suppression (11, 12). Indiscriminate use of antibiotics in severe trauma patients may significantly worsen their outcome (13), delay the diagnosis of a subsequent infection, and promote the emergence of multi-drug resistant microbes. Because of their similar clinical symptoms, it is crucial to implement precise and efficient diagnostic tools that allow fast and reliable differentiation between sterile inflammation and microbial sepsis in trauma patients. The study by Chung et al. showed that trauma patients developing sepsis (11.85% of the total cohort of trauma patients) deteriorated at a rate significantly higher than those who did not develop sepsis, 28.0% versus 4.6%, p >0.001 (14). This distinction is also critical for the development of timely surgical interventions (removal of infective foci in wounds, laparotomy for perforated bowel) and tailored antimicrobial treatments that can help improve patient outcomes (14, 15).

Trauma is the primary cause of mortality among individuals under the age of 45, with sepsis significantly contributing to these fatalities following traumatic injuries and infections (16). The incidence of sepsis among trauma patients is an alarming concern, where it significantly increases mortality (17). The annual incidence of sepsis in hospitalized patients is estimated to be over 750,000, with a mortality rate close to 30% (15). In trauma patients, a large percentage of deaths occur before reaching the hospital or within the first few hours after admission (15). Infections related to trauma often affect multiple organs and can manifest early, within the first four days. This holds true in trauma patients with contaminated wounds and fractures (18), early aspiration pneumonia in brain-injured patients (19), or superinfection of lung contusions (20). Some trauma patients may experience systemic inflammatory response syndrome (SIRS), while others may exhibit a compensatory anti-inflammatory response syndrome (CARS) (21), both leading to a modulated inflammatory response. The risk of infection in trauma patients arises from factors such as the disruption of mechanical barriers, bacterial contamination, local wound conditions, and invasive medical procedures (*e.g.* invasive mechanical ventilation, central venous lines, and catheters). Additionally, host defense factors can be compromised, leading to impaired immune responses (22). Infections secondary to trauma are most often of bacterial origin but can also be caused by viruses or, more rarely, by fungi (23).

Differentiating sterile inflammation from sepsis in trauma patients is crucial for appropriate management and treatment. The usually used parameters, C-reactive protein, CRP, and Interleukin-6, IL-6, are unfortunately stimulated via common mechanisms in both sterile and septic inflammation, and even procalcitonin (PCT) shows restrictions in that it is mainly induced by bacterial and not viral or atypical bacterial sepsis. According to Pierrakos et al. these parameters may rather be used to exclude sepsis (24). Recent findings confirm that PCT cannot accurately discriminate infectious vs. noninfectious acute inflammation in critically ill patients with severe traumatic injuries (area under the receiver operating characteristics curve: 0.53; 95% CI, 0.42–0.64) (25). In addition, machine learning algorithms were used to analyze a large multi-omic database of over 8500 markers, including proteomics, metabolomics, and lipidomics, to identify prognostic biomarkers in trauma patients’ admission plasma samples for predicting outcomes like mortality and recovery. Abdelhamid et al. thus revealed that a combination of five proteins was best for discriminating critical illness resolution and 26 multi-omic features were effective in predicting 30-day survival, with the study suggesting the potential for novel prognostic biomarkers in trauma patients’ admission data (26).

Early recognition of sepsis is essential for improving clinical prognosis and reducing mortality (15, 24, 27). The definition of sepsis has evolved over time, with the latest definition emphasizing life-threatening organ dysfunction caused by a dysregulated host response to infection (28, 29). Quick Sepsis-associated Organ Failure Assessment (qSOFA) scores are used as screening tools to aid in the rapid diagnosis of sepsis (28). However, their discriminative power in surgical intensive care patients is very low (9) and the challenge to identify patients at risk has resulted in new approaches for scores *e.g.* SAPS3 and biomarkers (30–32). Trauma patients thus present unique challenges (one of them being the timely and discriminate diagnosis of infection), and their clinical course can be complicated by sepsis and sepsis-induced mortality (33).

Timely diagnosis and treatment of infection in these patients are associated with improved outcomes and reduced mortality (34). However, the standard methods for diagnosing pathogens during sepsis, such as blood or bronchoalveolar lavage cultures, can be limited in sensitivity and turnaround time (35, 36). New diagnostic techniques are being explored to enhance pathogen detection in sepsis cases (35, 37). For instance, mass spectrometry has emerged as a promising tool for identifying biomarkers in the metabolome and proteome, potentially enhancing the early diagnosis and prognosis of sepsis (38). Identifying new biomarkers to differentiate between damage-induced sterile inflammation and sepsis in trauma patients could provide valuable insights for early detection of infection and timely intervention (39). Diagnostic tests that rapidly and reliably identify the presence or absence of infection in the ICU population, including severe trauma patients are a pressing unmet need (40). Biomarkers can help in identifying subclinical changes before the establishment of disease, allowing for early therapeutic interventions (41). However, further research is needed to establish the utility and reliability of such biomarkers in the diagnosis and management of trauma and sepsis patients (42). In the present study, we show that advanced technologies, including MALDI and MAA, can aid in identifying biomarkers to distinguish between early stage sepsis and non-infected inflammation in ventilated trauma patients.

## Patients and methods

### Statement of studies involving human subjects

Informed consent was obtained from the patients or their guardians included in this study. Samples from Helsingborg Hospital ICU were approved by the Ethics committee in Lund Dnr. 2014/195, 2015/467 and 2019/04558 and samples from Charles-University Prague were approved by the Ethics Committee of the Military University Hospital Prague Dnr. 108/9-36/2016-UVN. Samples from Strasbourg University Hospital were approved by the French Agence Nationale de la Sécurité du Médicament et des Produits de Santé (ANSM, on October 5, 2018) and a National Institutional Review Board (CPP, on November 6, 2018, CPPIDF1-2018-ND51-Cat. 1; N°IRB/IORG#: IORG 0009918), which covers all participant sites. No patient identifiable information is presented.

### Patient population

#### Trauma patients

The trauma population consisted in 23 ventilated trauma patients included in the Traumadornase multicenter study [NCT03368092 (43)] in the following study sites: Clermont-Ferrand, Reims and Strasbourg University Hospitals, all in France. As per protocol, these patients were adult severe trauma patients (Injury Severity Score [ISS (44)] > 15, under mechanical ventilation and included within the first 18 hours after admission to the trauma bay. Severe trauma patients under mechanical ventilation are more challenging than their non-ventilated counterparts as they develop more healthcare-associated infections, namely but not restricted to ventilation-associated pneumonia (VAP) and whose definitive diagnosis remains a serious challenge (45). Those patients had their blood sampled on days 1 (admission to the ICU), 3, and 5 and were screened daily throughout their ICU stay for the development of subsequent VAP according to the Center Disease Control and Prevention (46) and other infectious diseases requiring antimicrobial treatment according to the definition of the International Sepsis Forum (47). Infections in trauma patients were then adjudicated based on post-hospitalization review.

#### Sepsis patients

The sepsis group contained 23 patients with community acquired sepsis, included after admission to the ICU within 24 hours of arrival to the hospital in Helsingborg, Sweden. These sepsis patients were a subgroup of patients included in an earlier reported cohort *(* 48). Inclusion criteria were; non-pregnant adults >18 years old with no surgery or blood transfusion during the 7 preceding days, and an expected ICU stay > 2 days (49). All patients with community aquired sepsis were on mechanical ventilation and received vasopressors, all had positive blood cultures, and they were all defined as having septic shock (28). According to ICU routine, they were assessed by the Simplified Acute Physiology Score 3 (SAPS3) (50). Clinical scores and blood tests were obtained daily for seven consecutive days. Day 1 was the day of arrival in the ICU.

In both trauma and sepsis patients, blood samples were drawn into EDTA tubes and after a centrifugation step stored at −80°C until use. Plasma samples from even healthy volounteers were also collected.

### Enzyme assays

#### Multiplex antibody array

Using RayBiotech, Inc.’s Quantibody^®^ Technology Array service, inflammatory proteins were analyzed using a glass slide-based quantitative antibody array (Human L-507 and L-493) as described by the manufacture’s instructions.

#### Enzyme-linked immunosorbent assay

For measurements in plasma the following ELISA kits were used: TREM1 (Cat No: OKEH00303; Aviva Systems Biology), IL1F10 (Cat No: EKX-E3NR3R; Nordic BioSite), Serpin A9 (Cat No: MBS935459; MyBioSource), A2M (Cat No: EKX-6NKMQS; Nordic BioSite), GPI (Cat No: OKEH06383; Aviva Systems Biology), VIPR2 (Cat No: EKH3534; Nordic BioSite), S100A10 (Cat No: EKX-EWP59E; Nordic BioSite), IL3 (Cat No: EKH392; Nordic BioSite), SYT13 (Cat No: MBS9316889; MyBioSource) and TNFRSF10 (Cat No: OKEH04990; Aviva Systems Biology). Protein concentrations were measured by using human antigen specific ELISA Kits according to manufacturer’s protocols. Absorbance was measured in a VICTOR3™ Microplate Reader (Perkin Elmer) as described previously (51).

### High performance liquid chromatography and mass spectrometry

#### Sample preparation and digestion

The abundant plasma proteins were depleted using the High-Select™ Top12 abundant protein depletion resin kit (Thermo Fischer Scientific, Waltham, MA, USA) according to the manufacturer’s instructions. Briefly, an aliquot of 8 µl from each sample was transferred onto the conditioned immuno depletion resin and incubated at room temperature (RT) for 20 min. Depleted proteins were collected by centrifugation at 1000 × g for 2 min. SDS was added at a concentration of 2%. The samples were digested with trypsin using the filter-aided sample preparation (FASP) method (52). Briefly, the samples were reduced with 100 mM dithiothreitol at 60°C for 30 min. The reduced samples were transferred to 30 kDa MWCO Pall Nanosep centrifugation filters (Pall Corporation), washed several times with 8 M urea and once with digestion buffer (DB, 0.5% sodium deoxycholate in 50 mM TEAB) prior to alkylation with 10 mM methyl methanethiosulfonate in digestion buffer for 20 min in room temperature. Digestions were performed by addition of Pierce MS grade Trypsin (Thermo Fisher Scientific) in DB to a trypsin:protein ratio of 1:100 and incubated overnight at 37°C. The next morning, an additional portion of trypsin was added and incubated for another three hours at 37°C. Peptides were collected by centrifugation and labeled using TMT (tandem mass tag) 10-plex isobaric mass tagging reagents (Thermo Scientific) according to the manufacturer’s instructions. Labeled samples from each sample type were combined into two sets, and sodium deoxycholate was removed by acidification with 10% TFA. The combined TMT-labeled samples were desalted using Pierce Peptide Desalting Spin Columns (Thermo Scientific) following the manufacturer’s instructions.

#### Fractionation and nLC-MS/MS analysis

Each set of samples was pre-fractionated on the Dionex Ultimate 3000 UPLC system (Thermo Fischer Scientific) using the Waters XBridge BEH C18 column (3.0 mm x 150 mm, 3.5µm, Waters Corporation, Milford, USA) and the a linear gradient of solvent A and B was applied as follows - gradient from 3% to 40% solvent B over 18 min, from 40% to 100% B over 5 min, 100% B for 5 min, all at the flowrate of 0.4 ml/min. Solvent A was prepared with 10 mM ammonium formate in water at pH 10.0, solvent B was prepared with 90% acetonitrile, 10% 10 mM ammonium formate in water at pH 10.0. Solvent A was 0.2% formic acid and solvent B was 80% acetonitrile, 0.2% formic acid. The 40 primary fractions were concatenated into 20 fractions (1 + 21, 2 + 22, ..., 20 + 40), evaporated and reconstituted in 3% acetonitrile, 0.2% formic acid for nLC-MS/MS analysis. Each fraction was analyzed on an Orbitrap Fusion Tribrid mass spectrometer interfaced with an Easy-nLC 1200 nanoflow liquid chromatography system (both Thermo Fisher Scientific). Peptides were trapped on the Acclaim Pepmap 100 C18 trap column (100 μm x 2 cm, particle size 5 μm, Thermo Fischer Scientific) and separated on the in-house packed C18 analytical column (75 μm x 32 cm, particle size 3 μm) using the gradient from 5% to 32% B in 75 min, from 32% to 100% B in 5 min, and 100% B for 10 min at a flow of 300 nl/min. MS scans were performed at 120,000 resolution, m/z range 380-1200. MS/MS analysis was performed in a data-dependent manner, with a top speed cycle of 3 s for the most intense doubly or multiply charged precursor ions. Precursor ions were isolated in the quadrupole with a 0.7 m/z isolation window, with dynamic exclusion set to 10 ppm and a duration of 45 seconds. Isolated precursor ions were subjected to collision induced dissociation (CID) at 35 collision energy with a maximum injection time of 50 ms. Produced MS2 fragment ions were detected in the ion trap followed by multinotch (simultaneous) isolation of the top 7 most abundant fragment ions for further fragmentation (MS3) by higher-energy collision dissociation (HCD) at 60% and detection in the Orbitrap at 50 000 resolutions, m/z range 100-500.

#### Database search and quantification

MS raw data files for the TMT set were merged for relative quantification and feature identification conducted using Proteome Discoverer version 1.4 (Thermo Fisher Scientific). A database search for each set was performed with the Mascot search engine (Matrix Science) using the Homo Sapiens Swissprot database, version Mars 2017 with 553941sequences. MS peptide tolerance of 5 ppm and MS/MS tolerance for identification of 600 millimass units (mmu), tryptic peptides with zero missed cleavage and variable modifications of methionine oxidation, fixed modifications of cysteine alkylation, N-terminal TMT-label and lysine TMT-label were selected. The detected peptide threshold in the software was set to a significance of FDR 1% by searching against a reversed database and identified proteins were grouped by sharing the same sequences to minimize redundancy. For TMT quantification, the ratios of the TMT reporter ion intensities in HCD MS/MS spectra (m/z 126-131) from raw data sets were used. Ratios were derived by Proteome Discoverer using the following criteria: fragment ion tolerance as 3 mmu for the centroid peak with the smallest delta mass and a minimum intensity of 2000. Only peptides unique for a given protein were considered for relative quantitation, excluding those common to other isoforms or proteins of the same family. The quantification was normalized within Proteome Discoverer 1.4, using the global median of all proteins. Calculations of the ratios were made by using a reference sample made from a mix of 4 of the samples or the control sample as denominator.

#### Protein abundance and enrichment analysis

All analyses of Matrix-Assisted Laser Desorption/Ionization (MALDI) and multiplex antibody array (MAA) data were performed in the open-source programming environment R (www.r-project.org, version 4.2.1). The same approach for data processing was followed for both datasets, generated by MALDI (777 analytes) and MAA (995 analytes) screening. First, for each analyte the ratio of protein levels between the trauma and sepsis groups was computed. The log2 of the aforementioned ratio was then used for downstream analysis and for ranking analytes. To highlight the relative abundance of the top 100 ranked proteins contrasting the trauma and sepsis groups, heatmaps were created using the ‘ComplexHeatmap’ package (version 2.12.1).Venn diagrams displaying unique and common proteins among all time points from the previously selected batch were constructed with the ‘nVennR’ package (version 0.2.3). For the data from MALDI screening, considering the log2-fold change of all given proteins, pathway enrichment analysis was done utilizing the ‘clusterProfiler’ package (version 4.7.1.3). The minimal allowed set size included in the enrichment analysis was set to 5. For reproducibility of the results, a seed was set. Enriched KEGG pathway terms as well as the associated proteins were depicted in barplots and Gene-Concept Networks with the help of the ‘enrichplot’ package (version 1.18.4).

### Statistical analysis

ELISA data were processed and presented with Microsoft Excel 2021 (Microsoft, Redmond, WA) and GraphPad Prism 9.0 (GraphPad Software, San Diego, CA). All results are presented as mean values ± SEM with the number of independent experiments and patients per group indicated in the figure legends. The comparison of data was performed by one-way ANOVA, Tukey’s multiple comparison test and area under the curve (AUC) analysis were performed with 95% confidence interval, using the Wilson/Brown method.

## Results

### Populations under study

Demographic, clinical and infectious data for the 23 included trauma patients are given in **Table 1**. In brief, these were predominantly young, male, severe trauma ICU patients mechanically ventilated for a median duration of 7 days. Among the trauma cohort, 3 patients developed VAP on days 1, 3 and 6, respectively (day 0 being admission day). No patient in the trauma cohort developed any infectious disease (excluding VAP) requiring antimicrobials during the first seven days. As depicted in **Table 2**, patients in the sepsis cohort were older, comorbid ICU patients in whom the sources of infection were mainly the respiratory tract and soft tissue. Biological workup and invasive therapeutic procedures (renal replacement therapy, invasive mechanical ventilation, vasopressor infusion) reflect the moderate severity of these septic ICU patients. SOFA scores in the trauma and sepsis populations (8 [5,5-10] and 11 [8,5-101, respectively) were non-significantly different (p=0.14). The plasma samples from 7 healthy individuals were used as controls.

**Table 1:** Demographic, clinical and infectious data in trauma patients.

Characteristics n=23	Median or n	Interquartile or percentage	Missing data
Age (years)	45	[25,5-55,5]	0
Male sex	20	87%	0
Glasgow Coma Scale on field	6	[3-13,5]	0
ISS	30	[26-39,5]	0
SOFA score on admission	8	[5,5-10]	0
Mechanical ventilation > 48h	23	100%	0
Transfused within 7 days (n)	18	78%	0
Number of blood products transfused	6	[1-14,5]	0
Duration of mechanical ventilation (hours)	117	[41-334]	0
ICU length of stay (hours)	245	[122,5-690,5]	0
Hospital length of stay (days)	31	[17,5-41]	0
Alive on day 28 (n)	20	87%	0
Developed VAP (CDC criteria, n)	3	13%	0
Developed any infectious disease (excluding VAP) requiring antimicrobials (n)	0	0%	0
Developed ARDS (n)	1	4%	1
Developed MOF^1^ (n)	1	4%	0

^1^Multi-organ failure (MOF) was defined as a SOFA score of 3 or more in at least two organ systems, assessed daily during the first days (53, 54).

**Table 2:** Demographic, clinical and infectious data in sepsis patients.

Characteristics n=23	Median or n	Interquartile or percentage	Missing data
Age (years)	72	[60-75]	0
Male sex	13	56,5%	0
Weight (kg)	74,5	[68-104]	1
Comorbidities:			0
° Cardiovascular disease	14		
° Heart failure	5		
° Chronic kidney disease	4		
° Hypertension	10		
° Diabetes	7		
° Liver disease	1		
° COPD	7		
° Malignancy	1		
SOFA score on admission	11	[8,5-11]	0
SAPS III	69	[61,5-74]	0
Source of infection			0
° Unknown	3		
° Abdomen	2		
° Respiratory tract	8		
° Urogenital	3		
° Skin/Soft tissue	7		
Renal replacement therapy (n)	3	13%	0
Invasive mechanical ventilation > 48h (n)	11	48%	0
Vasopressors (n)	22	96%	0
Peak CRP concentration (mg/L)	220	[156-335]	0
Peak PCT concentration (µmol/L)	31	[10-101]	0
Peak lactate concentration (mmol/L)	2.6	[1.3-3.45]	0
Peak SOFA score	11	[8,5-14]	0
Alive on Day 28 (n)	22	96%	0

### Representation of protein abundance and enrichment analysis

**Figure 1:**
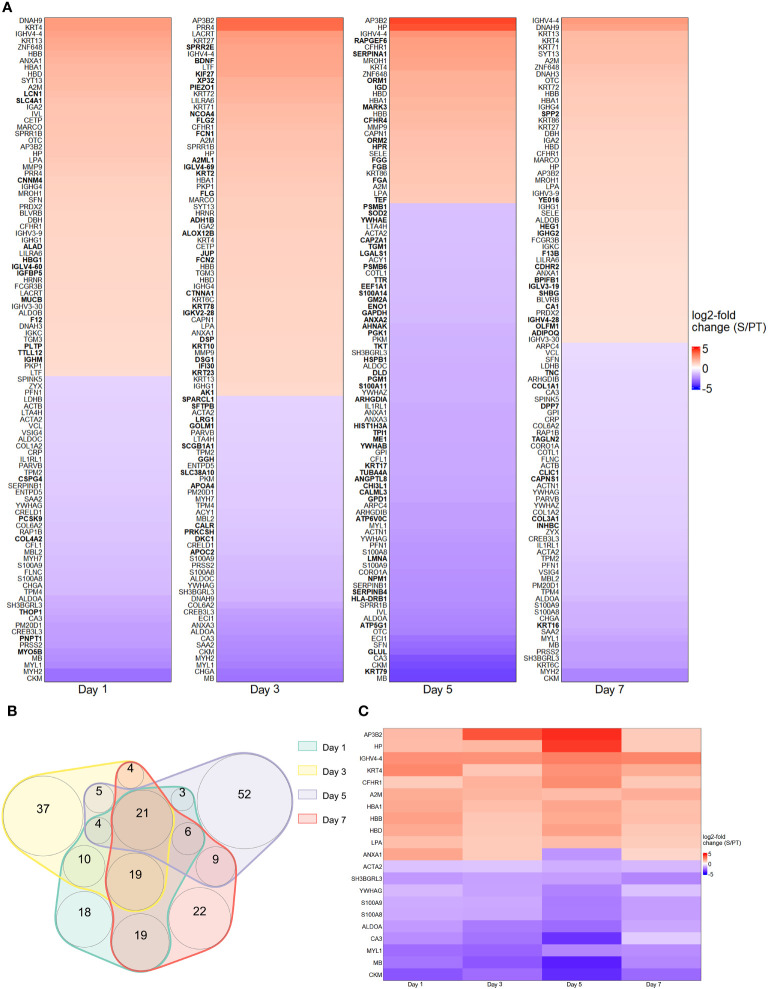
Protein abundance after MALDI screening. **(A)** Top 100 differentially regulated proteins at day 1, 3, 5 and 7 contrasting trauma and sepsis groups. The relative abundance of protein levels is shown as log2-fold change from sepsis to trauma (S/PT) group. Protein names that occurred only on a single day were printed in bold. **(B)** Venn diagram showing the common and unique proteins among all time points. **(C)** Heatmap highlighting the 21 recurring proteins on all days.

**Figure 2:**
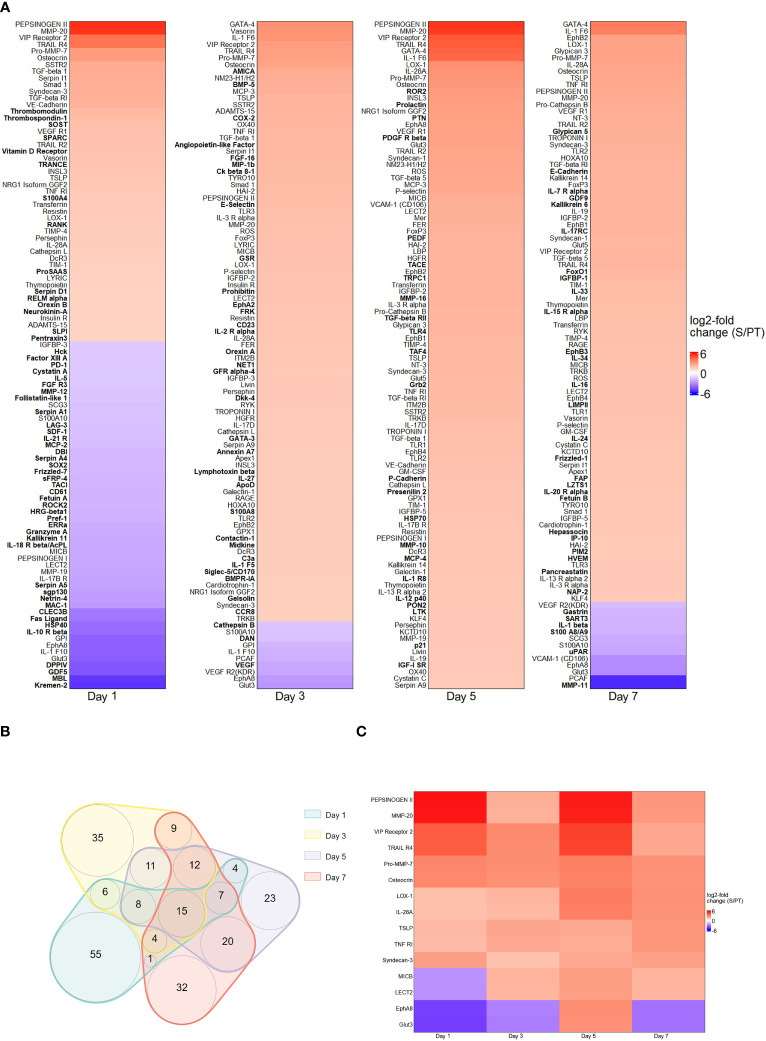
Protein abundance after MAA screening. **(A)** Top 100 differentially regulated proteins at day 1, 3, 5 and 7 contrasting trauma and sepsis groups. The relative abundance of protein levels is shown as log2-fold change of sepsis to trauma (S/PT) group. Protein names that occurred only on a single day were printed in bold. **(B)** Venn diagram showing the common and unique proteins among all time points. **(C)** Heatmap highlighting the 15 recurring proteins on all days.

The first series of experiments was performed to investigate the protein abundance in plasma samples from trauma and sepsis patients. To achieve this goal, we utilized cutting-edge technologies such as MALDI and MAA, respectively. These two methods allowed us to probe the proteomic terrain, unveiling specific biomarkers that exhibit potential for early detection and differentiation of trauma and sepsis patients. By applying comparative analysis methods, we assessed the protein levels in plasma sampled from patients with trauma and sepsis. To illustrate their expression patterns, the top 100 proteins (ranked by their log2 fold change) derived from the MALDI and MAA screenings are presented in **Figures 1A**, **2A**. The log2-fold changes within the sepsis to trauma (S/PT) group spanned a range from approximately 5.6 to -5.4 and 4.4 to -4.0 within the 7-day time period tested, respectively. Positive numbers indicate higher protein levels in the sepsis group, while negative numbers present higher protein levels in the trauma group. Among the selected proteins, the distribution of common and unique biomarkers at different time points was depicted with Venn diagrams (**Figures 1B**, **2B**). A total of 15 and 21 proteins were revealed to recur on all days, and their relative abundance was singled out in an additional heatmap (**Figures 1C**, **2C**).

**Figure 3:**
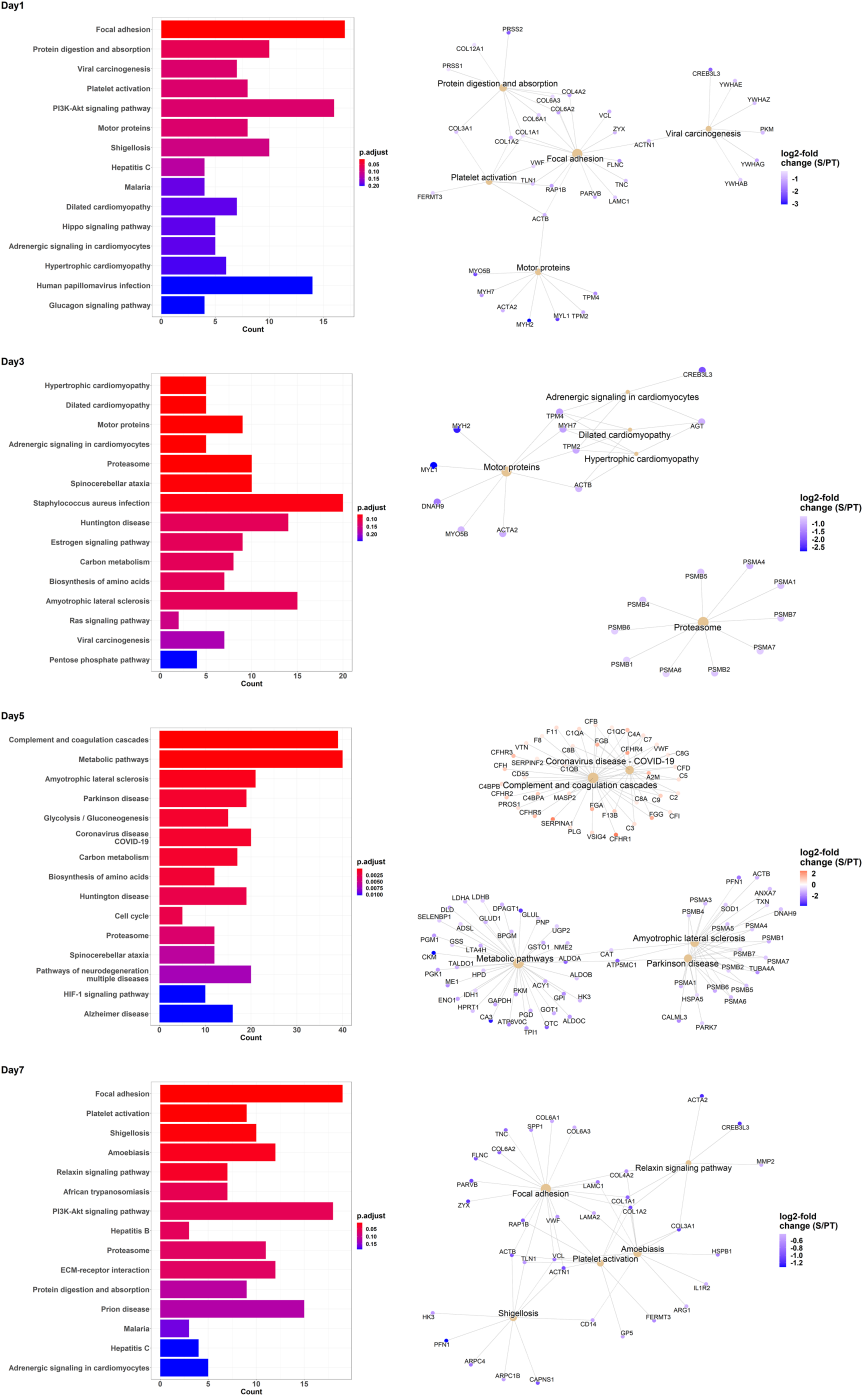
Enrichment analysis of KEGG pathway terms for differentially regulated proteins contrasting trauma and sepsis group at various days. The top 15 KEGG pathway terms are listed for the days 1, 3, 5 and 7. Additionally, Gene-Concept Networks illustrate the relevant proteins associated with the top 5 KEGG pathway terms per time point. The relative abundance of protein levels is shown as log2-fold change of sepsis to trauma (S/PT) group.

We also performed an enrichment analysis on the MALDI data independently for each day. **Figure 3** shows the top 15 enriched KEGG pathways per day, together with Gene-Concept Networks illustrating the top 5 enriched terms featuring the associated proteins and their relative abundance. While proteins belonging to the complement and coagulation cascades (see day 5) were generally raised, others, such as proteins relevant for focal adhesion (see day 1, day 7), were mostly diminished in the sepsis group compared to the trauma group. In summary, the comparative analysis of protein abundance and enrichment patterns between trauma and sepsis patients reveals distinct molecular signatures. In sepsis, elevated complement and coagulation cascade proteins contrast with diminished focal adhesion-related proteins, underscoring their potential as crucial diagnostic indicators.

### Identification of potential biomarkers

**Figure 4:**
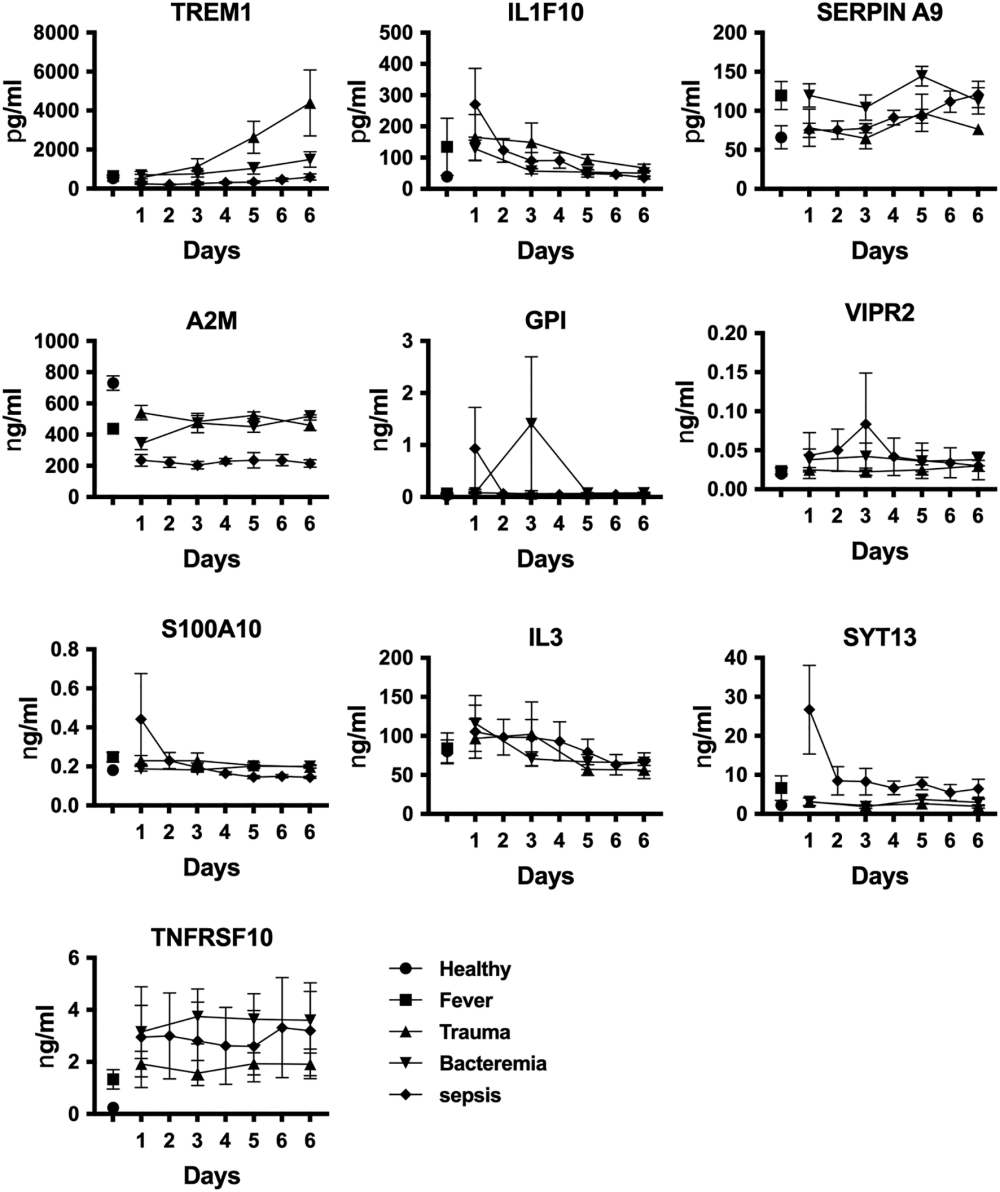
*Identification of sepsis biomarkers.* We assessed plasma protein levels using quantitative ELISA at various stages of disease development. Data are displayed as mean +/- SEM. Healthy individuals (n=6), fever (n=6), bacteremia (n=5), trauma (n=4), and sepsis (n=6).

The next series of experiments aimed at identifying potential biomarkers to distinguish sepsis patients from individuals with other inflammatory conditions. This proof-of-concept study involved comparing protein levels in various scenarios, including fever (n=6), bacteremia (n=5), trauma (n=4), and sepsis (n=6), using plasma samples from healthy individuals (n=6) as controls (**Supplementary Table 1**). To authenticate candidate biomarkers, we conducted a comprehensive analysis and identified IL1F10, A2M, GPI, VIPR2, S100A10, and SYT13 as potential targets (**Figures 1**, **2**). Plasma protein levels were also assessed at various time points using ELISA (**Figure 4**). Additionally, we explored the measurement of other noteworthy targets, namely IL3 (55), TREM1 (56), SERPIN A9 (57), and TNFRSF10 (58) (**Figure 4**). Our analysis showed enhanced levels of SYT13, IL1F10, and S100A10 cytokines in early sepsis patients, while TREM1 levels in trauma patients increased gradually over time (**Figure 4**). Notably, A2M levels were consistently lower in inflammatory circumstances, particularly in sepsis patients. Additionally, we observed the stability of SERPIN A9, GPI, IL3, VIPR2, and TNFRSF10 levels across different conditions, revealing their limited potential as discriminatory biomarkers (**Figure 4**).

### Profiling biomarkers to differentiate between trauma and sepsis patients

**Figure 5:**
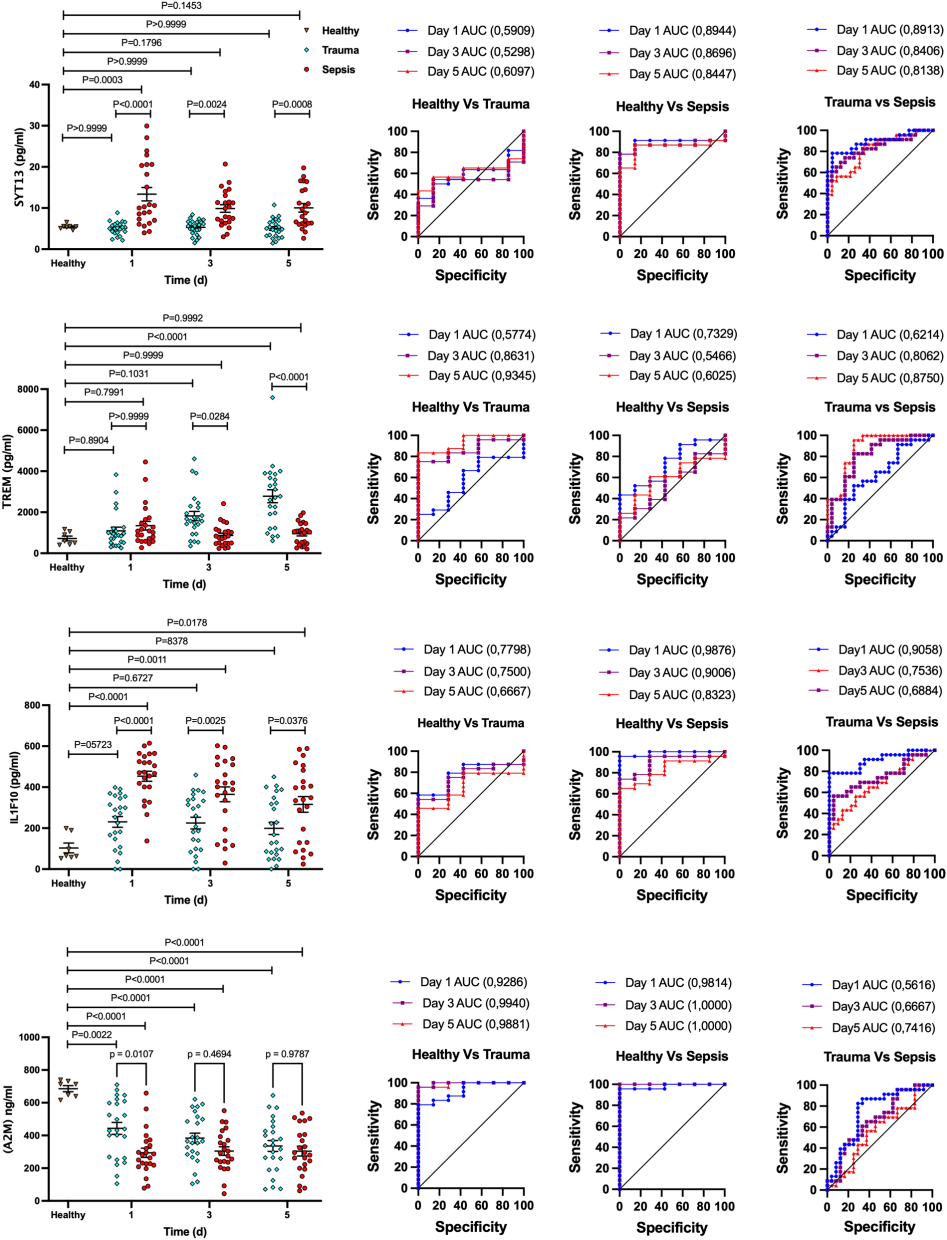
*Potential biomarker panel to distinguish trauma and sepsis patients.* Quantitative ELISA was used to determine the levels of A2M, IL1F10, SYT13, and TREM1 in trauma and sepsis patients at various time periods. The comparison of data was performed by one-way ANOVA, Tukey’s multiple comparison test and area under the curve (AUC) analysis were performed with 95% confidence interval, using Wilson/Brown method. Healthy (n=7); trauma ans sepsis (n=23).

To validate our findings, we extended the number of plasma samples for trauma (n = 23) and sepsis patients (n = 23) and performed quantitative ELISA to measure the contents of A2M, IL1F10, SYT13, and TREM1 in these samples at three time points (day 0, 3 and 5). Plasma samples from healthy individuals (n = 7) were used as control. This analysis spanned various time points in both trauma and sepsis cases, covering the initial (day 0) and later stages (day 5) of the ICU stay. As seen before, distinct patterns emerged with increased SYT13 and IL1F10 levels in plasma samples from sepsis patients, while A2M levels were decreased when compared to the level detected in plasma samples from trauma patients (**Figure 5**). TREM1 levels, however, increased only in plasma samples from trauma patients but not in those from sepsis patients over the course of time (**Figure 5**). These findings confirm that the identified biomarkers, A2M, IL1F10, SYT13, and TREM1, hold significant potential in distinguishing trauma and sepsis patients. In addition, the data was further tested using ROC curves (**Figure 5**, right). For early detection of sepsis patients from trauma, the AUC for SYT13 is 0.8913 and for IL1F10 is 0.9058. As a result of the findings, SYT13 and IL1F10 can be employed as diagnostic biomarkers with high prediction efficiency.

**Figure 6:**
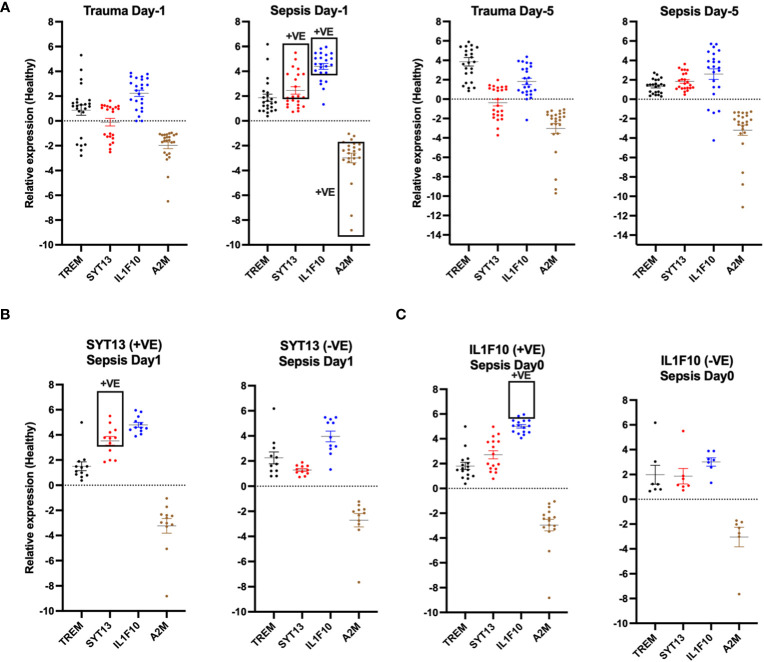
*SYT13 and IL1F10 differentiate trauma and sepsis patients.* **(A)** The levels of the proteins A2M, IL1F10, SYT13, and TREM1 at the early (day 0) and later stage (day 5) of the infection. **(B)** A comparison of the protein levels of SYT13 at high and low concentrations [+Ve; positive; from **(B)**] with those of A2M, IL1F10, and TREM1. **(C)** Comparison of the A2M, SYT13, and TREM1 and protein levels with the high and low protein levels of IL1F10 [+Ve; positive; from **(B)**]. Data are displayed as mean +/- SEM. Healthy (n=7); trauma and sepsis (n=23).

To validate our results, we compared the relative expression levels of the four proteins with samples from healthy individuals at both early (day 0) and later time points (day 5). We set a minimum relative 2-fold shift in the expression levels as an assessment threshold. Using this setting, we measured clear distinctions between the biomarker profiles of trauma and sepsis patients. At the early time point, SYT13 and IL1F10 displayed a minimum 2-fold shift increase in expression levels in sepsis patients when compared to trauma patients (**Figure 6A**). Additionally, A2M showed a minimum 2-fold shift decrease in sepsis patients (**Figure 6A**). Further analysis focused on the correlation of these biomarkers within early sepsis samples. We observed that SYT13-positive samples showed a positive correlation with IL1F10 (**Figure 6B** left). Interestingly, IL1F10 positivity was also observed in SYT13-negative samples (**Figure 4C** right), suggesting the complexity of biomarker interactions in sepsis cases.

A similar analysis compared early sepsis samples with positive and negative IL1F10 2-fold shift outcomes. Our results showed that IL1F10-positive samples correlated positively with A2M while displaying limited associations with SYT13 or TREM1 (**Figure 6C** left). Conversely, TREM1-negative samples exhibited negligible correlations with other biomarkers (**Figure 6C** right).

To conclude, our research emphasizes the potential of A2M, IL1F10, SYT13, and TREM1 as a composite set of biomarkers for distinguishing between non-infected trauma and sepsis patients. Detailed information about the biological functions of the four proteins is provided in **Table 3**. The clear distinctions in their expression patterns offer a pathway for accurate and early diagnosis, necessitating further investigation to validate their clinical applicability.

**Table 3:** Identified biomarkers and their biological function.

Protein	Biological Function	Reference
A2M	A plasma protein that inhibits various proteases and binds to cytokines, growth factors, and hormones	(59–62)
IL1F10 or IL38	A cytokine that modulates inflammatory responses and immune cell activation	(63–66)
SYT13	A synaptic vesicle protein that regulates neurotransmitter release and membrane trafficking	(67–69)
TREM	A family of receptors that mediate innate immune responses and regulate inflammation, tissue homeostasis, and cancer	(70–74)

## Discussion

In this study, we aimed at identifying a potential biomarker panel that can differentiate trauma-induced sterile inflammation from sepsis. Both emergencies constitute severe medical conditions with overlapping clinical manifestations, making their early and accurate differentiation challenging. To address this diagnostic problem, we employed advanced technologies, including Matrix-Assisted Laser Desorption/Ionization (MALDI) and multiplex antibody arrays (MAA), to profile the protein abundance in trauma and sepsis patients at different time points.

Our results revealed distinct protein patterns associated with the initial stages of trauma and sepsis. A2M, IL1F10, SYT13, and TREM1 emerge as compelling biomarkers for sepsis and trauma based on their distinct roles in immune response modulation. A2M is identified as a biomarker in trauma and sepsis cases, emphasizing its involvement in anti-inflammatory responses and tissue repair, making it a promising indicator for trauma-related diagnostics (60). IL1F10 belongs to the interleukin-1 (IL-1) family of cytokines (75) and in particular IL1F10 has been suggested being of diagnostic and prognostic value as clinical sepsis biomarker (76). The role of SYT13 in sepsis still unknown, whereas TREM1 is a crucial mediator of septic shock that acts by synergizing with Toll-like receptors (TLRs) to amplify the inflammatory responses to pathogens, thus promoting sepsis-induced immune dysregulation and organ dysfunction (74). While the levels of TREM1 are found up-regulated during the course of the disease we found a negative correlation for SYT13 and IL1F10 which implicate that their downregulation during the course of infection may negatively impact the host response to infection.

In trauma and sepsis patients, the inflammatory response appeared to differ, and complement and coagulation cascades were generally increased in sepsis patients compared to trauma patients. On the other hand, proteins involved in focal adhesion were mostly diminished in sepsis patients, indicating a possible difference in immune response between the two conditions.

Our findings suggest that a combination of multiple biomarkers will be more effective in distinguishing sepsis patients from those with trauma-induced sterile inflammation. Indeed, multidimensional variability reduces the accuracy of single biomarker assays (77). Utilizing a panel of biomarkers may enhance diagnostic accuracy and provide valuable insights for early prediction of infection and timely and discriminate use of antimicrobials in trauma patients. The identified biomarkers could aid in the development of novel diagnostic methods to enable rapid and accurate identification of trauma-induced sterile inflammation and sepsis, facilitating early intervention and improving patient outcomes.

### Limitations

Deciphering infection from non-infective Systemic Inflammatory Response Syndrome (SIRS) remains a challenging task. This is all the more true as infections contributes to tissue injury and injury predisposes to infection (78). It is therefore crucial to acknowledge that our study has specific limitations associated with the patient populations under investigation. Even if our patient sample size compares favorably with previous publications in the field (25, 79, 80), its modest number warrants further confirmation in larger, at best multicentric, cohorts of both trauma and sepsis patients.

While our study provides a promising proof of concept, further research is needed to validate these findings in larger patient cohorts and across different healthcare settings. The use of additional patient samples and validation studies will be crucial to establishing the utility and reliability of the identified biomarker panel. The aspect of gene polymorphism in patients reflects upon the response to infection, *e.g* interferon γ (81), making diagnostic and therapeutic considerations even more complex, though this has not been an aim of the present study.

It is worth noting that the trauma cohort included in our study consisted only of patients requiring mechanical ventilation for at least 18 hours (L21). This inclusion criterion may introduce a level of selection bias, as it focuses on a subset of trauma patients with more severe respiratory compromise. Therefore, the generalizability of our findings to the broader trauma patient population may be limited.

Also all septic patients in our study suffered from septic shock (L40,41), representing a more severe form of sepsis. While this selection facilitates the identification of septic patients, including those with a more critical condition, it may limit the extrapolation of our results to less severe septic cases. The biomarkers and mechanisms identified in patients with septic shock might differ from those in patients with milder forms of sepsis.

Moreover, it is essential to note that our primary focus was on identifying biomarkers in trauma patients who developed sepsis. The mechanisms and biomarkers associated with sepsis development following trauma may differ from those in patients who develop sepsis due to other underlying diseases, as described elsewhere in this manuscript. This distinction is crucial, as the heterogeneity of sepsis etiology could impact the generalizability of our findings to diverse septic patient populations.

Despite these specific patient population considerations, our study offers a valuable approach to distinguish trauma patients who develop sepsis from those who do not. The identified biomarkers show promise in aiding the early detection of infection in trauma patients. However, further research, including validation studies in larger and more diverse patient cohorts, is warranted to confirm the clinical utility and generalizability of the identified biomarker panel.

## Conclusion

In conclusion, our proof-of-concept study highlights the potential of employing a biomarker panel for early differentiation of severely ill sepsis patients from those with trauma-induced sterile inflammation. By utilizing advanced technologies and exploring the inflammatory response in these patients, we identified specific proteins with diagnostic relevance. Implementing such a biomarker panel in clinical practice could significantly improve the early detection and management of infection in trauma patients, leading to better patient outcomes.

## Data availability statement

The datasets presented in this study can be found in online repositories. The names of the repository/repositories and accession number(s) can be found in the article/**Supplementary Material**.

## Ethics statement

The studies involving humans were approved by Ethics committee in Lund Ethics Committee of the Military University Hospital Prague French Agence Nationale de la Sécurité du Médicament et des Produits de Santé. The studies were conducted in accordance with the local legislation and institutional requirements. Written informed consent for participation was not required from the participants or the participants’ legal guardians/next of kin in accordance with the national legislation and institutional requirements. The studies were conducted in accordance with the local legislation and institutional requirements. The human samples used in this study were acquired from some of the co-authors involved in this study. Written informed consent for participation was not required from the participants or the participants’ legal guardians/next of kin in accordance with the national legislation and institutional requirements.

## Author contributions

PP: Conceptualization, Data curation, Formal analysis, Funding acquisition, Investigation, Methodology, Project administration, Resources, Software, Validation, Writing – original draft, Writing – review & editing. MS: Formal analysis, Software, Validation, Writing – review & editing. NP: Resources, Writing – review & editing. VL: Resources, Writing – review & editing. BR: Resources, Writing – review & editing. JO: Resources, Writing – review & editing. CR: Resources, Writing – review & editing. EB: Resources, Writing – review & editing. MH: Resources, Writing – review & editing. KJ: Conceptualization, Writing – original draft, Writing – review & editing, Resources, Validation. JP: Conceptualization, Formal analysis, Resources, Validation, Writing – original draft, Writing – review & editing. HH: Conceptualization, Funding acquisition, Project administration, Supervision, Writing – original draft, Writing – review & editing.

## Supplementary material

Supplementary Table 1 Demographic and clinical data.

Supplementary Table 2 Antibody microarray analysis data.

Supplementary Table 3 MALDI-Tandem Mass Tag (TMT) analysis data.
