# Supplementary material for: Identifying biomarkers deciphering sepsis from trauma-induced sterile inflammation and trauma-induced sepsis
Source: Front Immunol. 2024 Jan 12;14:1310271. doi: 10.3389/fimmu.2023.1310271 (PMC10820703; doi:10.3389/fimmu.2023.1310271)
Supplement: Supplementary Table 1 — Demographic and clinical data. [file Table_1.docx]

| **Supplemental Table -1** | **Sepsis** | **Bacteremia** | **Polytrauma** | **Fever** |
| --- | --- | --- | --- | --- |
|  | n=6 | n=5 | n=4 | n=6 |
| Age, Mean (SD) | 66 (13) | 62 (18) | 46 (18) |  |
| Sex, Male (%) | 5 (83) | 0 (0) | 3 (75) |  |
| Ethnicity, Caucasian (%) | 6 (100) | 5 (100) | 4 (100) |  |
| Weight, Mean (SD) | 84 (17) | 65 (11) | 89 (13) |  |
| Height, Mean (SD) | NA | 163 (4) | 176 (7) |  |
| BMI, Mean (SD) | NA | 24 (4) | 28 (4) |  |
| Lenght of hospital stay, Mean (SD) | # | 7 (1) | 64 (42) |  |
| Comorbidities |  |  |  |  |
| Diabetes mellitus (%) | 1 (17) | 1 (20) | 1 (25) |  |
| Cardiovascular (%) | 4 (67) | 3 (25) | 1 (25) |  |
| Chronic obstructive pulmonary disease (COPD) (%) | 1 (17) | 1 (20) | 0 (0) |  |
| Renal dysfunction (%) | 1 (17) | 0 (0) | 1 (25) |  |
| Malignancies (%) | 0 | 0 (0) | 1 (25) |  |
| Source of infection |  |  |  |  |
| Lung (%) | 3 (50) |  |  |  |
| Skin/tissue (%) | 2 (37) |  |  |  |
| Urinary tract | 1 (17) |  |  |  |
|  |  |  |  |  |
| # 3 sepsis patients stayed >7 days in ICU, total LOS unknown | | | | |
